# Supplementary material for: Duration of Initial Viremia Modulates Functional Properties of HIV-specific T Cell Receptors
Source: bioRxiv. 2026 Feb 2:2026.01.29.702605. Preprint. [Version 1] doi: 10.64898/2026.01.29.702605 (PMC12889509; doi:10.64898/2026.01.29.702605)
Supplement: Supplement 1 [file NIHPP2026.01.29.702605v1-supplement-1.pdf]

bioRxiv preprint doi: https://doi.org/10.64898/2026.01.29.702605; this version posted February 2, 2026. The copyright holder has placed this preprint (which was not certified by peer review) in the Public Domain. It is no longer restricted by copyright. Anyone can legally share, reuse, remix, or adapt this material for any purpose without crediting the original authors.

Supplementary Materials:

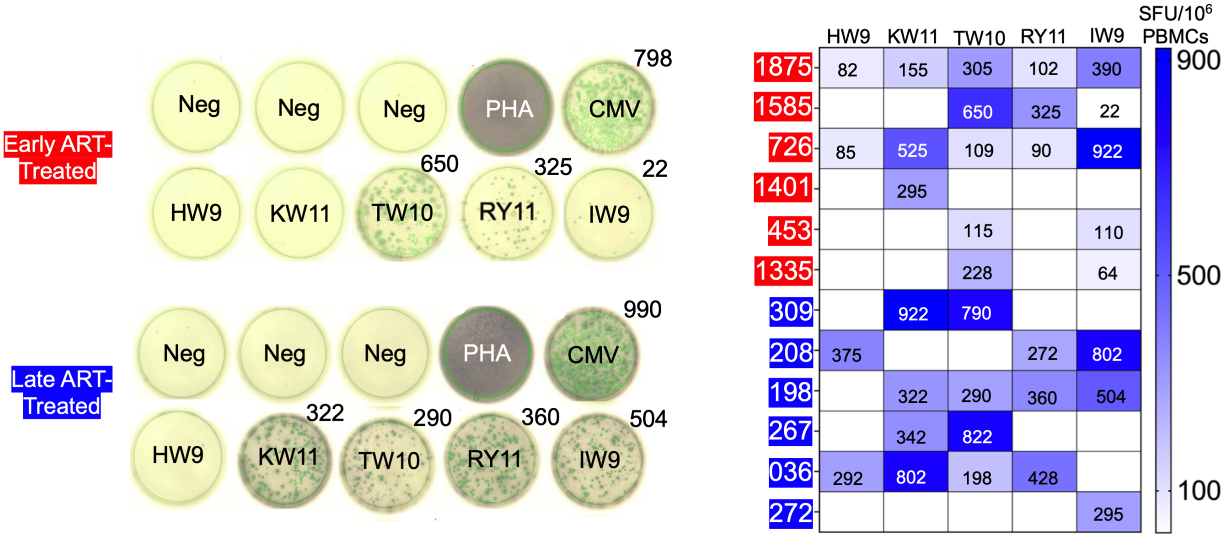

Figure S1: HLA-B*58:01-restricted HIV-specific CTL responses *ex vivo* and following *in vitro* expansion: Breadth and magnitude of HIV-specific CTL responses in PBMCs, measured by IFN-g ELISPOT to five clade C HLA-B*58:01-restricted consensus epitopes (TW10, IW9, KW11, RY11, and HW9) in the 12 participants, measured during the acute phase of HIV-1 infection. Reported values represent spot-forming units (SFUs) after subtraction of background activity from triplicate negative control wells without peptide stimulation[33]. Each column corresponds to an individual epitope, with response magnitudes depicted by a color scale. Numbers within each box indicate SFUs per 10^6 PBMCs. The intensity scale shown on the right denotes the strength of responses above background. Early and late treated persons are indicated by identifiers shown in red and blue boxes, respectively.

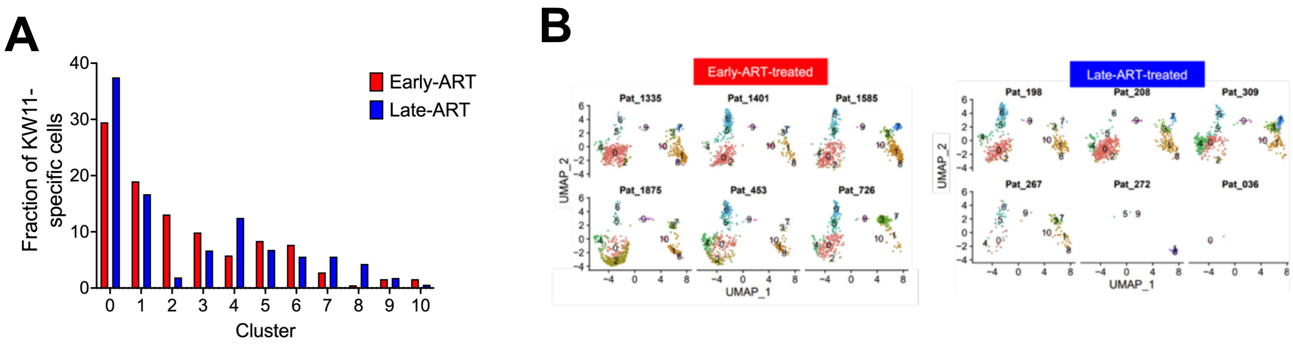

Figure S2: Comparison of KW11-specific clonotypes captured *ex vivo*. (**A**) Bar graphs showing the proportion of KW11-specific CD8$^+$ T cells from each cluster in Figure 6B. Red bars represent early ART-treated donors, and blue bars represent late ART-treated donors. (**B**) Cluster distribution by donor, showing the relative fraction of cells per participant, with donors 272 and 036 having limited cell recovery.

bioRxiv preprint doi: https://doi.org/10.64898/2026.01.29.702605; this version posted February 2, 2026. The copyright holder has placed this preprint (which was not certified by peer review) in the Public Domain. It is no longer restricted by copyright. Anyone can legally share, reuse, remix, or adapt this material for any purpose without crediting the original authors.

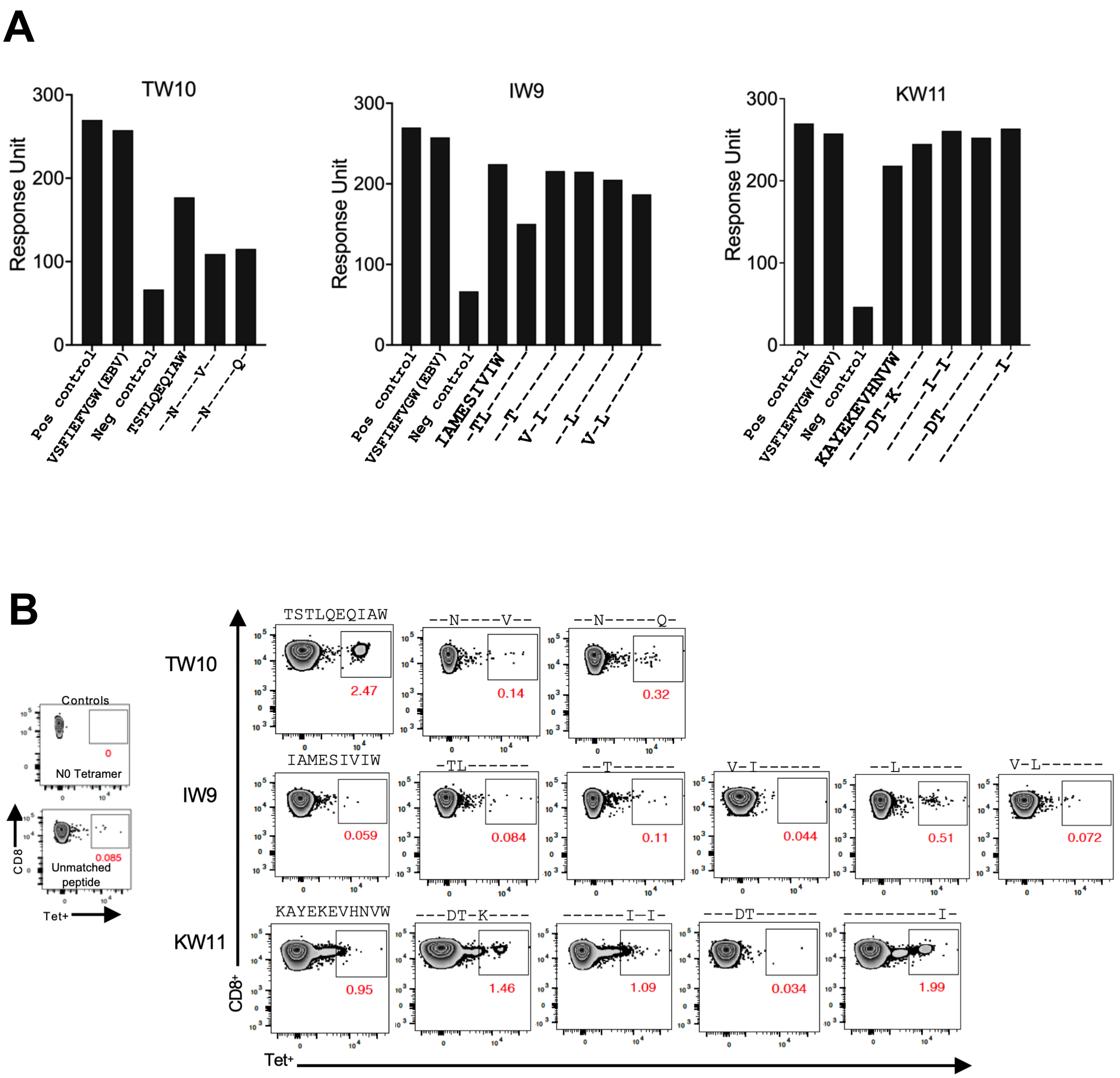

<u>Figure S3</u>: Validation of barcoded tetramer folding using Flex-T ELISA assay: (A) The bar graphs show response units from Flex-T sandwich ELISA assay, utilized to verify the correct folding of each barcoded tetramer. This assay confirmed proper folding for most barcoded tetramers, except for the TW10 variants, consistent with the stability results reported in this study. **(B)** Barcoded tetramers were used to stain $CD8^+$ T cells following expansion with soluble CD3/CD28, followed by a 2-day resting period. Representative plots are shown from one of the late-treated donors.

bioRxiv preprint doi: https://doi.org/10.64898/2026.01.29.702605; this version posted February 2, 2026. The copyright holder has placed this preprint (which was not certified by peer review) in the Public Domain. It is no longer restricted by copyright. Anyone can legally share, reuse, remix, or adapt this material for any purpose without crediting the original authors.

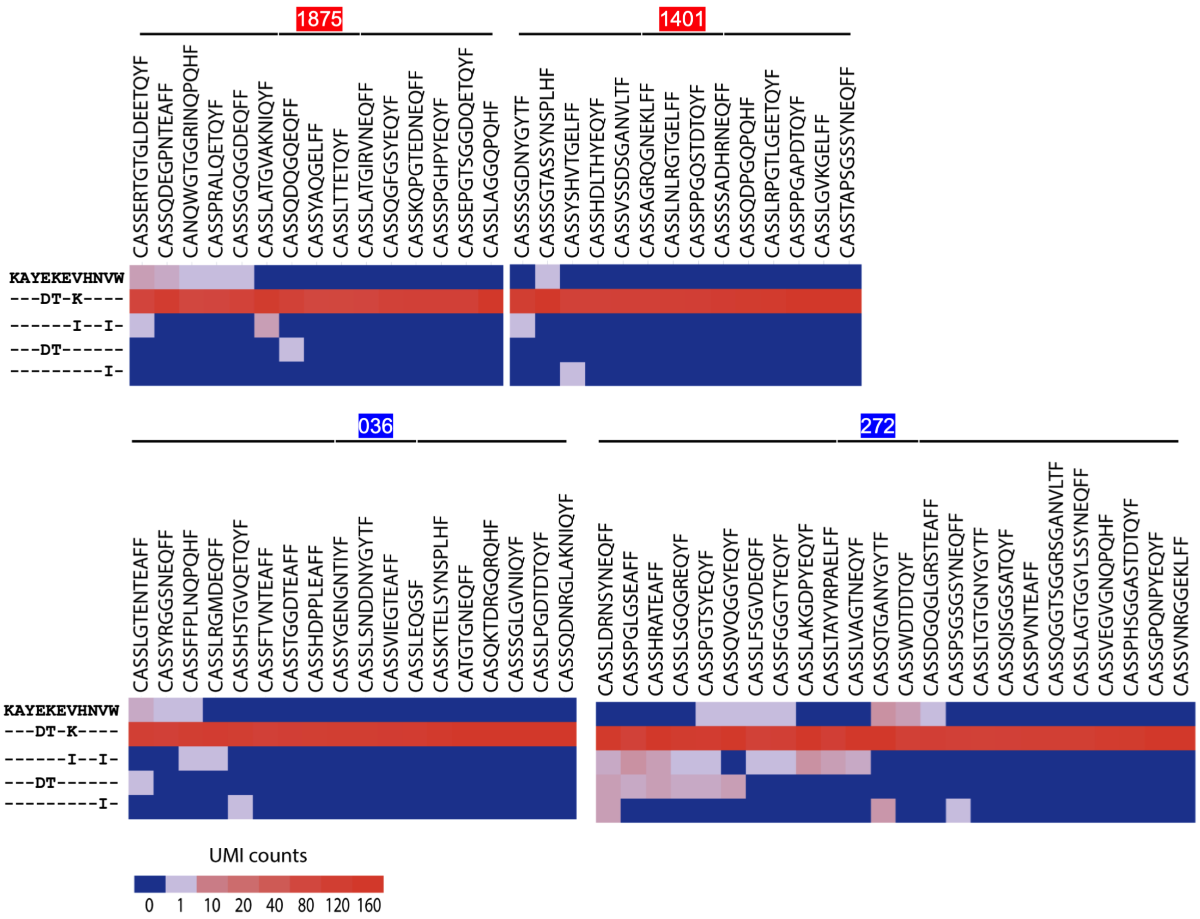

Figure S4: Representative heatmaps from two early treated (1875 and 1401) and two late-treated (036 and 272) participants, illustrating examples of the intensity of tetramer UMI counts per cell, stratified by assigned epitope-specificity. Several of the clonotypes shown here correspond to thise included in the Figure 4. Data are shown for the KW11 epitope using pooled, barcoded pHLA tetramers encompassing both consensus and variant KW11 epitopes. All epitopes used for pHLA tetramers were obtained from HLA-B*58:01-positive donors. Color intensity reflects increasing tetramer UMI counts for each clonotype, annotated by its CDR3β sequence. Clonotypes were considered positive if detected with ≥5 counts.

bioRxiv preprint doi: https://doi.org/10.64898/2026.01.29.702605; this version posted February 2, 2026. The copyright holder has placed this preprint (which was not certified by peer review) in the Public Domain. It is no longer restricted by copyright. Anyone can legally share, reuse, remix, or adapt this material for any purpose without crediting the original authors.

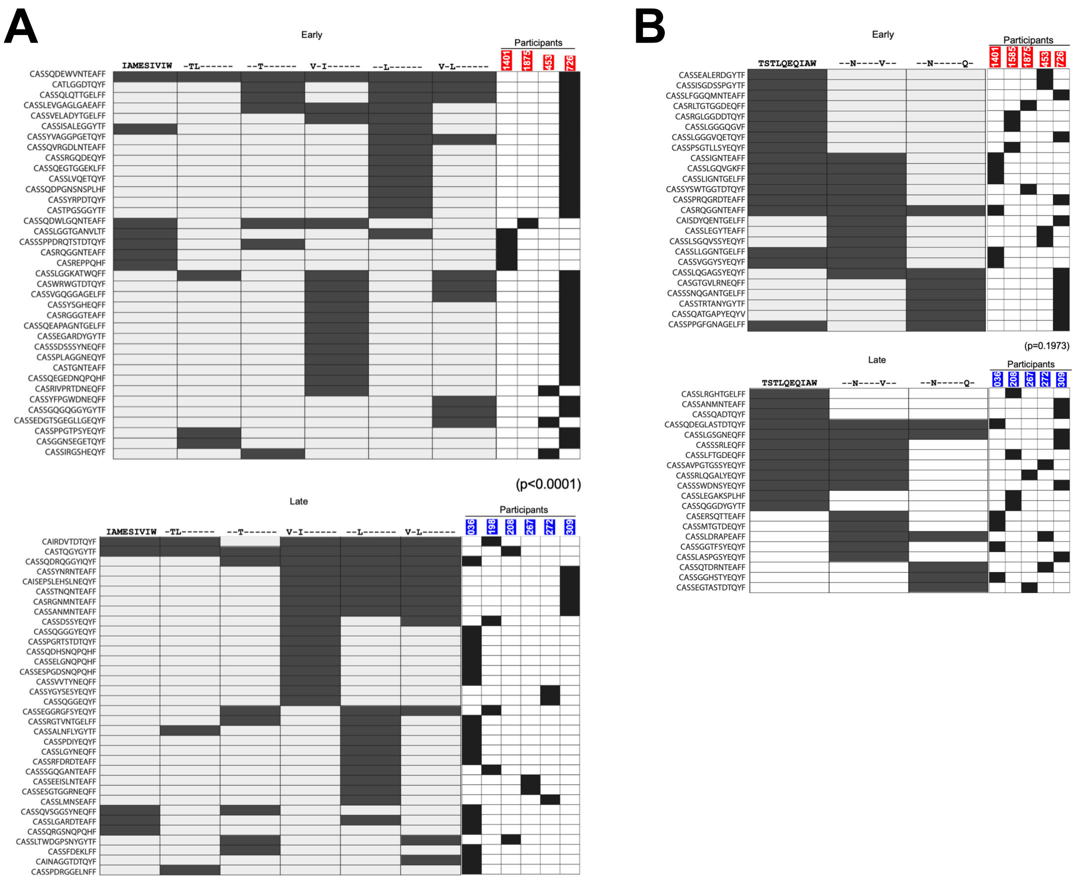

<u>Figure S5</u>: HIV-specific TCRs recognition in early versus late treated groups. Heatmaps illustrating cross-reactive TCR clonotypes targeting (**A**) the IW9 and (**B**) the TW10 antigen family. Clonotypes were detected in donors with immediate (top) and delayed ART-treatment initiation. Each heatmap shows TCR recognition and binding strength across consensus and variant epitopes. The right column indicates the relative contribution of individual donors within each treatment group to the observed cross-reactive pattern. For the IW9 epitope, 66% of IW9-specific clonotypes from early ART-treated individuals were monospecific, compared with 55% in late ART-treated individuals. For the TW10 epitope, 57% of TW10-specific clonotypes from early ART-treated individuals were monospecific, compared with 60% in late ART-treated individuals are not shown in these monochromatic panels.

bioRxiv preprint doi: https://doi.org/10.64898/2026.01.29.702605; this version posted February 2, 2026. The copyright holder has placed this preprint (which was not certified by peer review) in the Public Domain. It is no longer restricted by copyright. Anyone can legally share, reuse, remix, or adapt this material for any purpose without crediting the original authors.

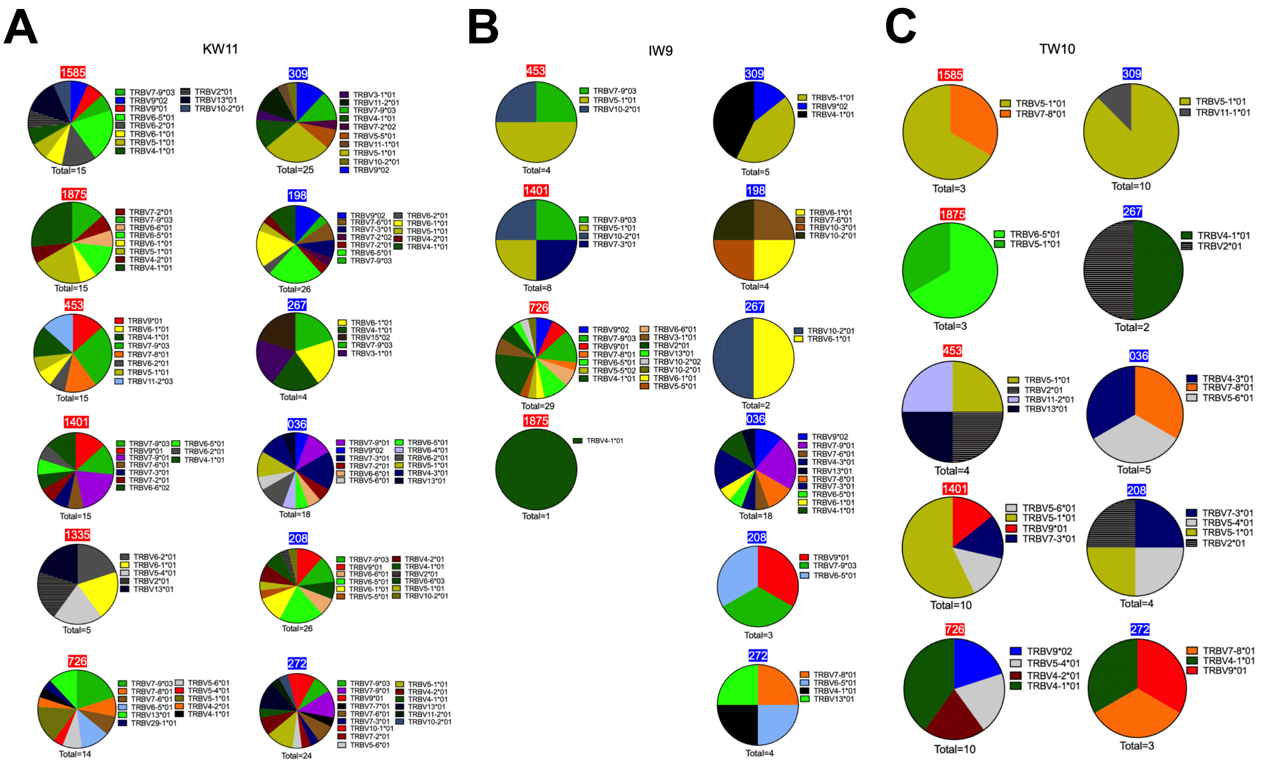

<u>Figure S6:</u> Shared TRBV gene usage among HIV-specific TCRs from early and late ART-treated individuals. (**A**) KW11, (**B**) IW9, and (**C**) TW10-specific clonotypes are shown. Each pie chart summarizes the proportion of TRBV genes usage within individual donors, with columns grouped by treatment category for each epitope. Donor IDs are indicated above each pie chart, with early ART-treated donors shaded in red and late ART-treated shaded in blue. The “total” value below each pie chart denotes the number of unique clonotypes detected. Pie slice size represents the relative frequency of clonotypes using each TRBV gene within that donor, with color coding to the right for each pie chart.

bioRxiv preprint doi: https://doi.org/10.64898/2026.01.29.702605; this version posted February 2, 2026. The copyright holder has placed this preprint (which was not certified by peer review) in the Public Domain. It is no longer restricted by copyright. Anyone can legally share, reuse, remix, or adapt this material for any purpose without crediting the original authors.

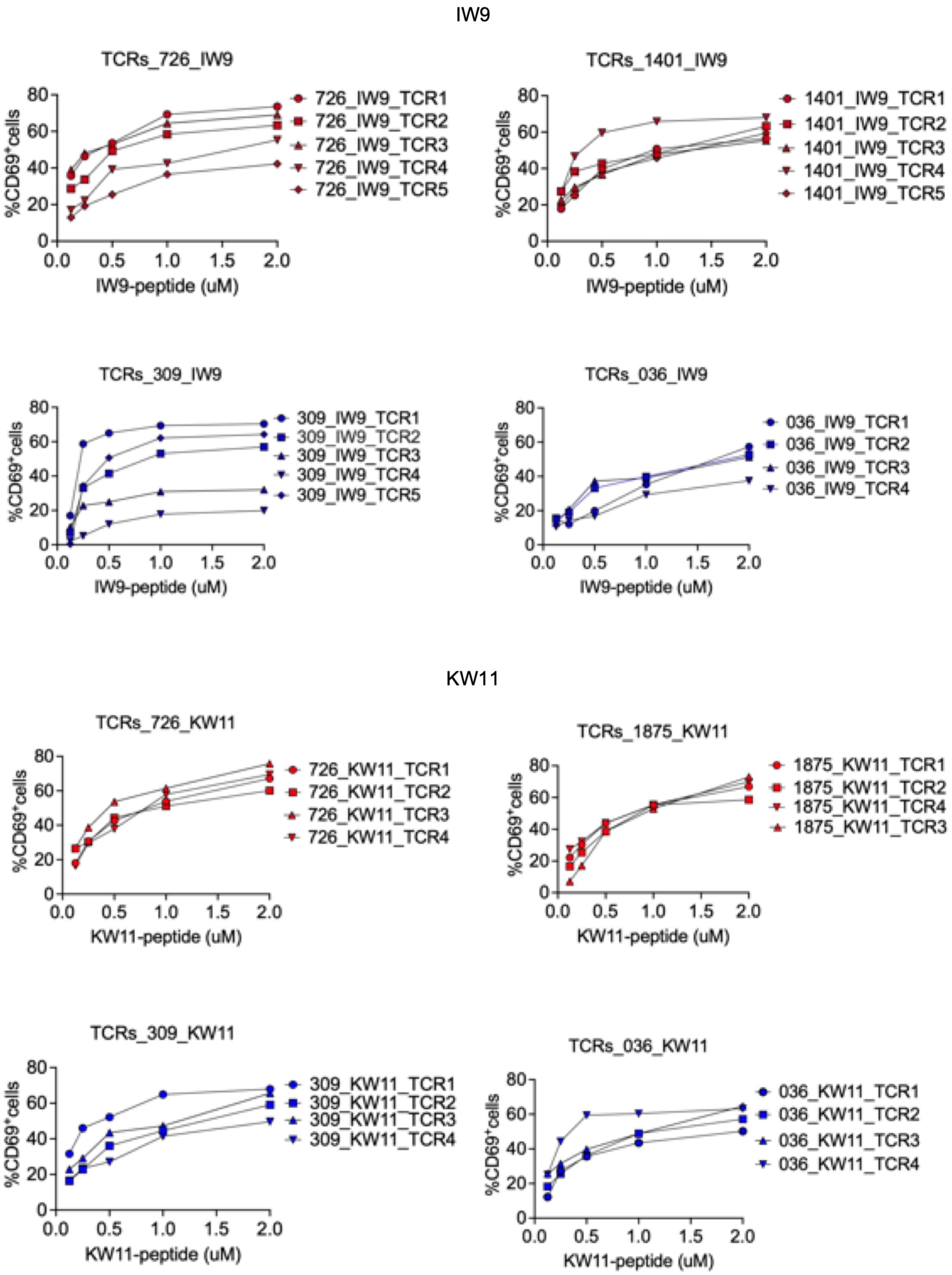

Figure S7: Normalized dose-response curves showing CD69 upregulation in monoclonal TCR-expressing Jurkat cells stimulated with IW9 (top) or KW11 (bottom) peptides. The Y-axis indicates the % of $CD69^+$ Jurkat cells, and the X-axis represents peptide concentration. Each curve corresponds to an individual TCR, labeled by its respective ID. Red curves denote TCRs derived from early-treated donors, while blue curves represent TCRs from late-ART-treated

bioRxiv preprint doi: https://doi.org/10.64898/2026.01.29.702605; this version posted February 2, 2026. The copyright holder has placed this preprint (which was not certified by peer review) in the Public Domain. It is no longer restricted by copyright. Anyone can legally share, reuse, remix, or adapt this material for any purpose without crediting the original authors.

donors. Peptide dose-response curves for each TCR were generated to estimate antigen sensitivity, with EC_{50} serving as a quantitative proxy for TCR functional responsiveness.

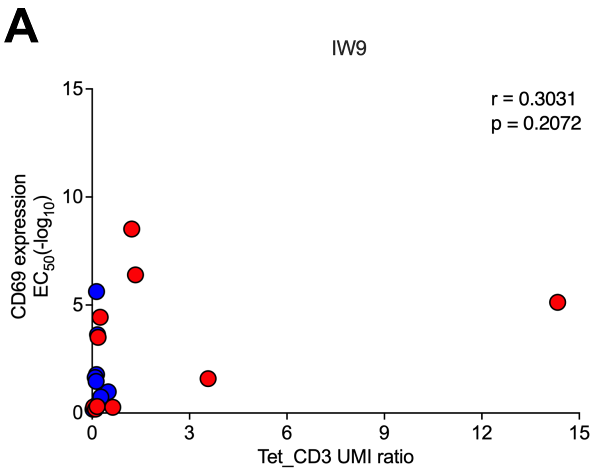

Figure S8: Relationship between peptide dose-responses and tetramer/CD3 UMI ratio. (**A**) IW9 and (**B**) KW11-specific TCRs. The EC_{50} for CD69 expression profile from peptide dose-response assays for each clonotype is on the y axis, and the corresponding tetramer/CD3 UMI ratio on the x-axis. Red dots indicate early ART-treated donors, and blue dots indicate late ART-treated donors.

Supplementary Table 1: HLA class I genotypes of study participants

Participant ID	HLA class I type
1401	A*2,29; B*15,58:01; Cw*3,
1335	A*2,74; B*50,58:01; Cw*6,7
1585	A*23,30; B*15,58:01; Cw*6,18
453	A*1,30; B*42,58:01; Cw*6,17
1875	A*2; B*45,58:01; Cw*7,16
726	A*30,33; B*53,58:01; Cw*4,6
267	A*23,74; B*35,58:01; Cw*4,6
309	A*2; B*58:01; Cw*7
208	A*23,68; B*8,58:01; Cw*3,7
198	A*23,30; B*15,58:01; Cw*3,16
036	A*2,30; B*44,58:01; Cw*4,6
272	A*30,68; B*18,58:01; Cw*2,3
